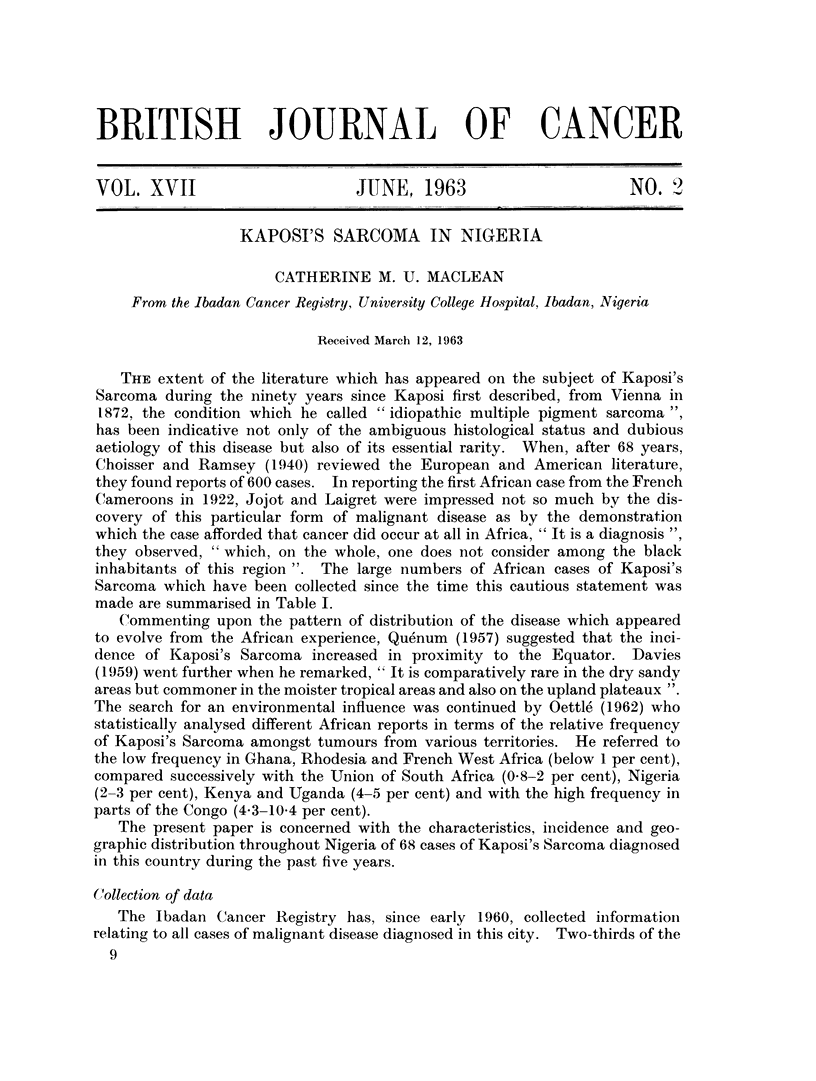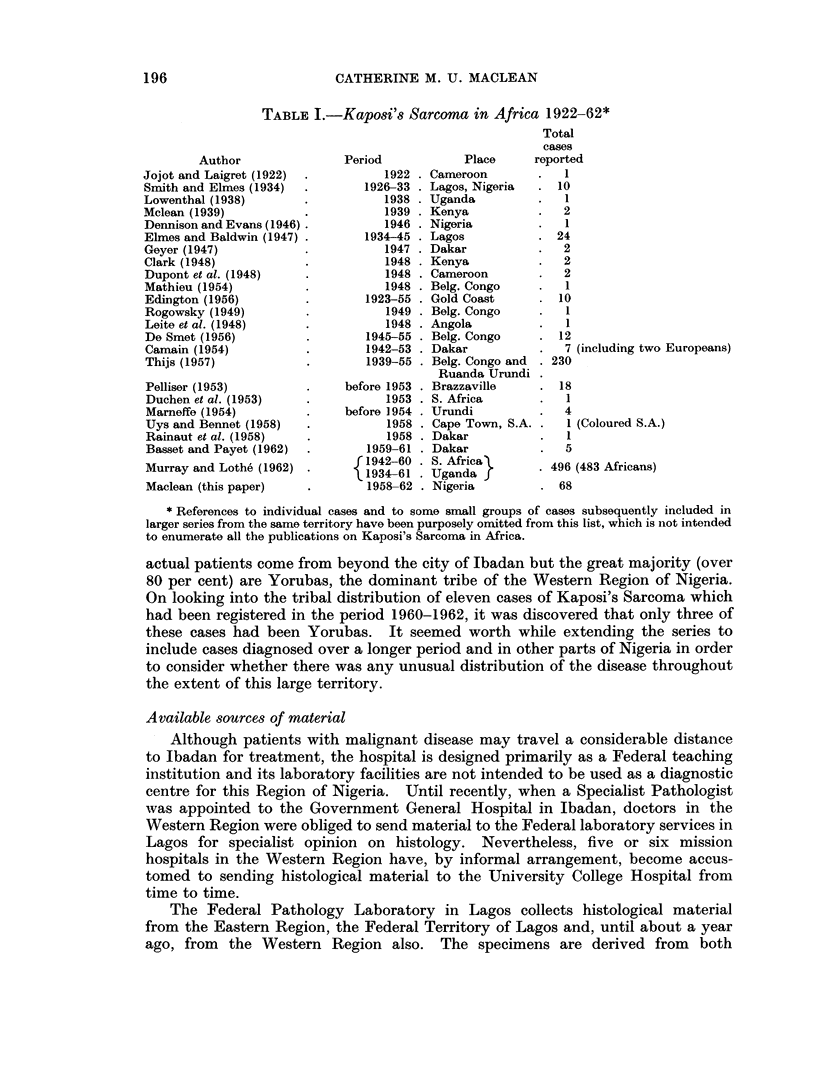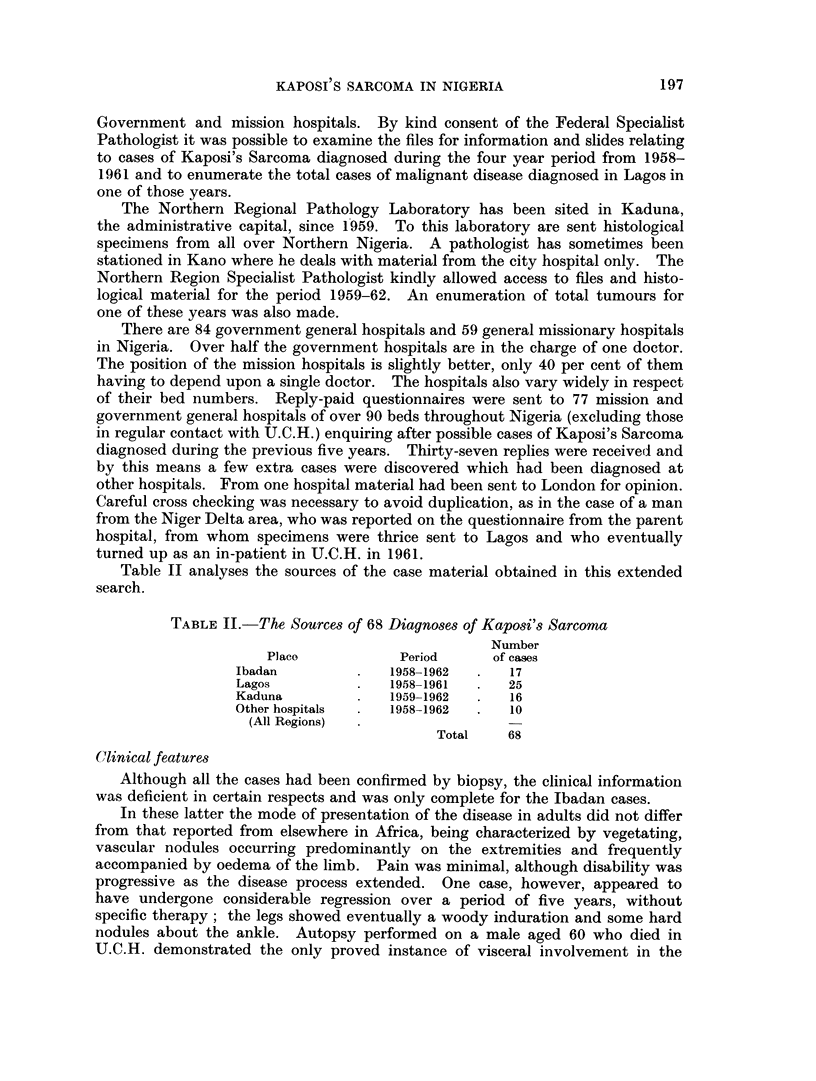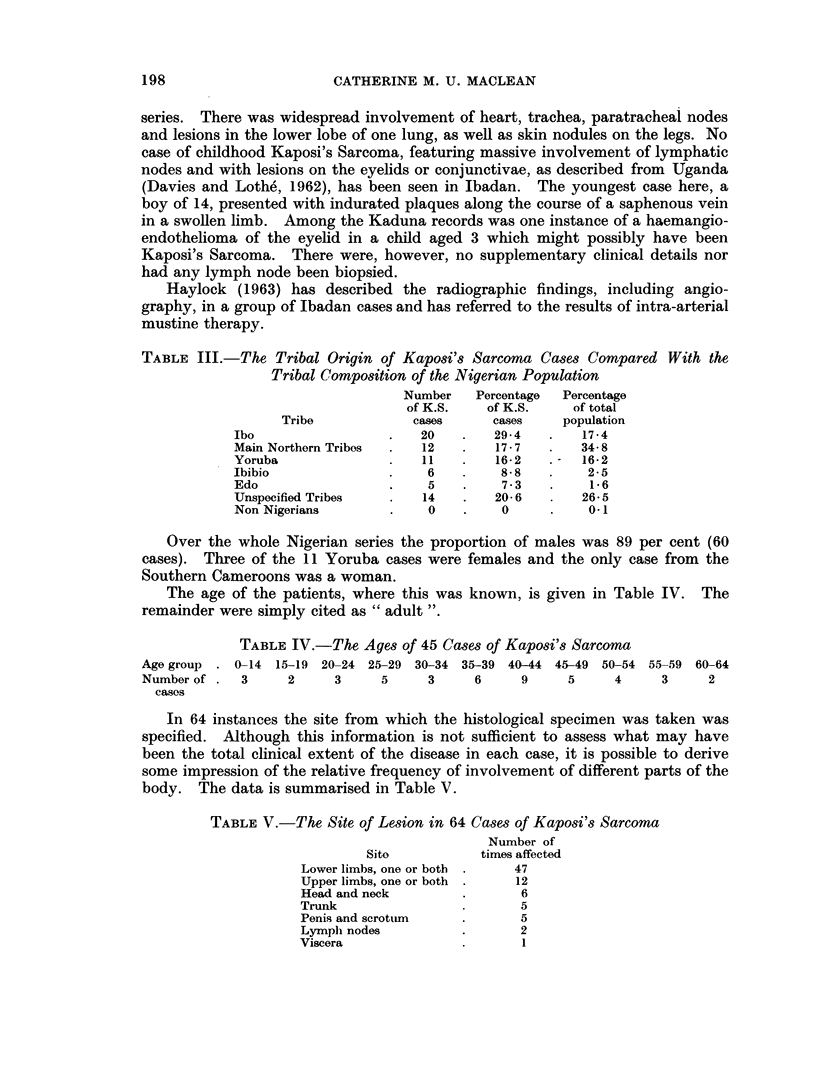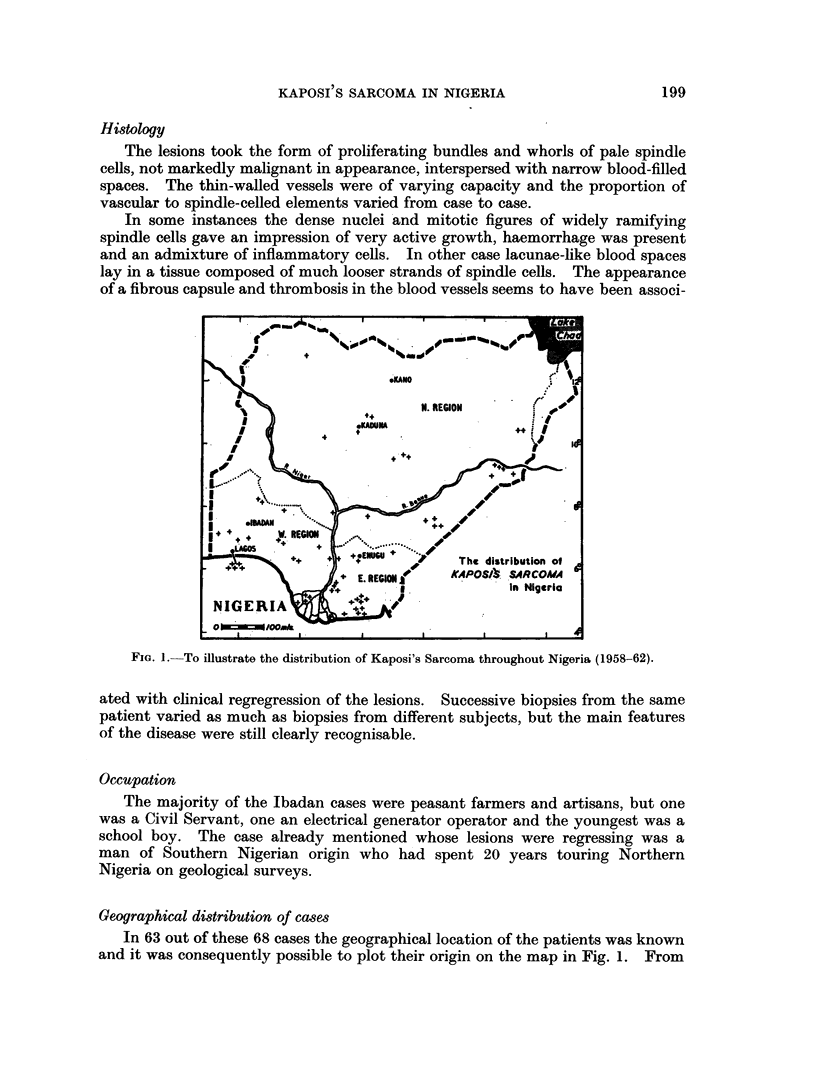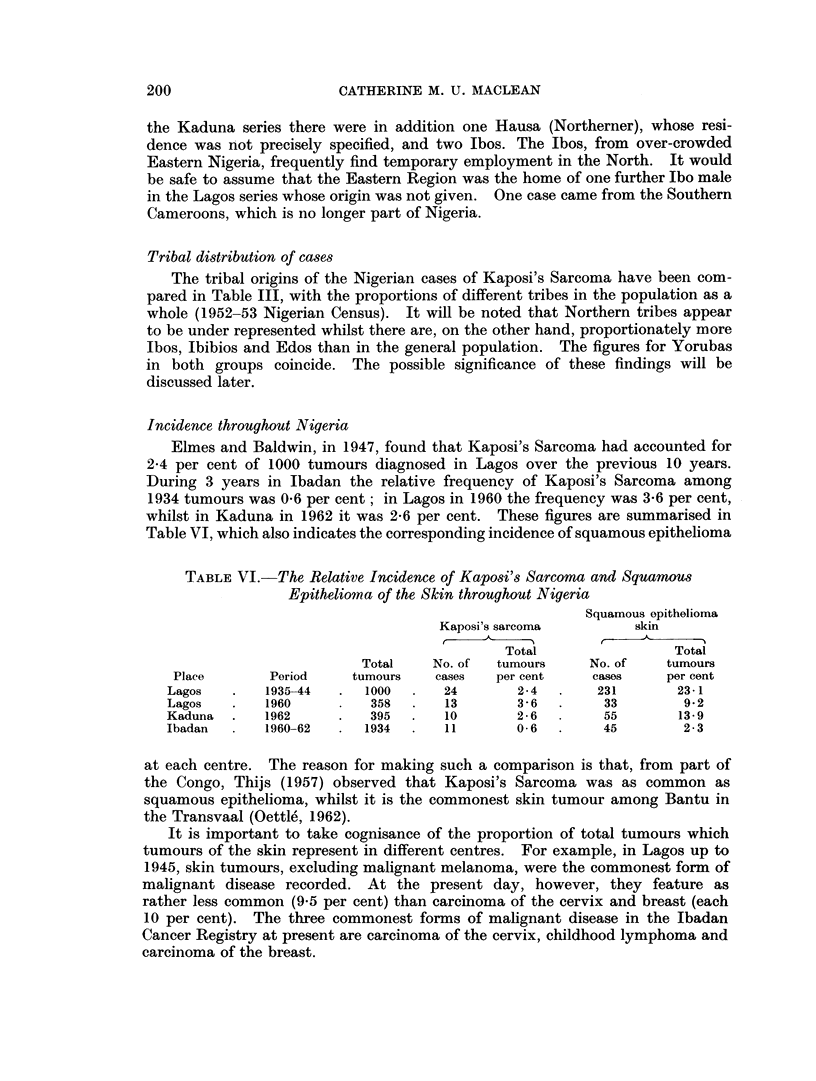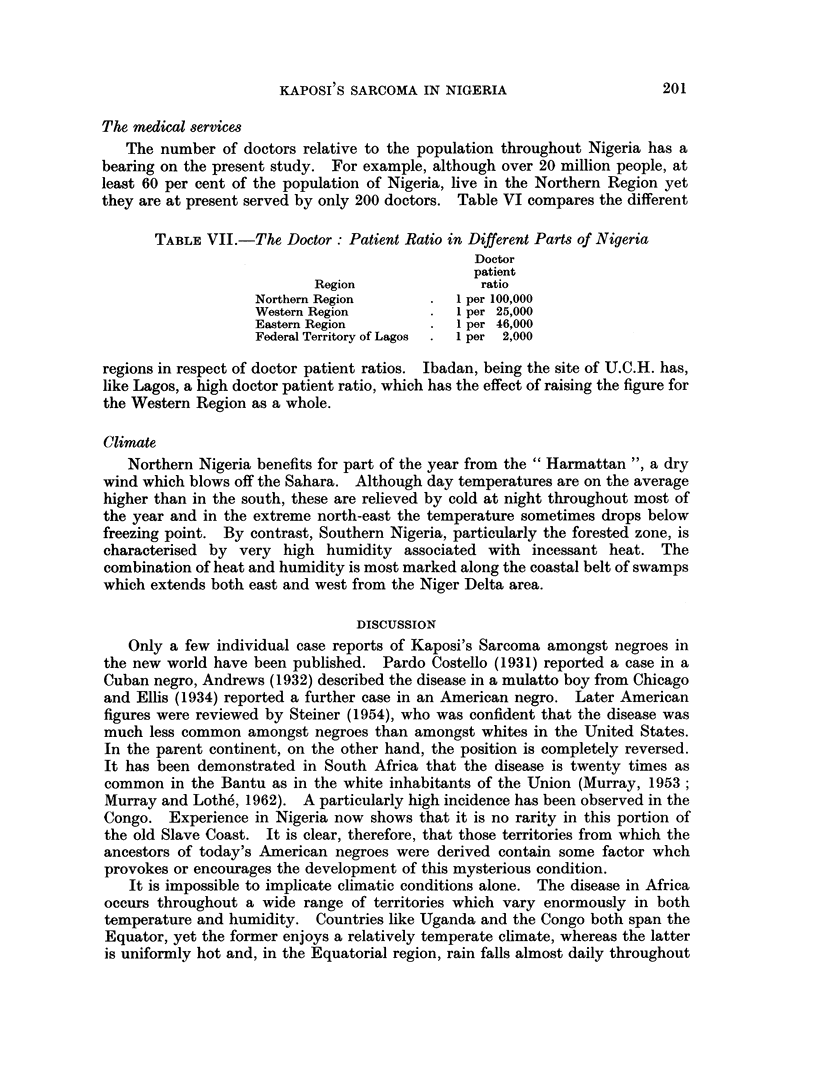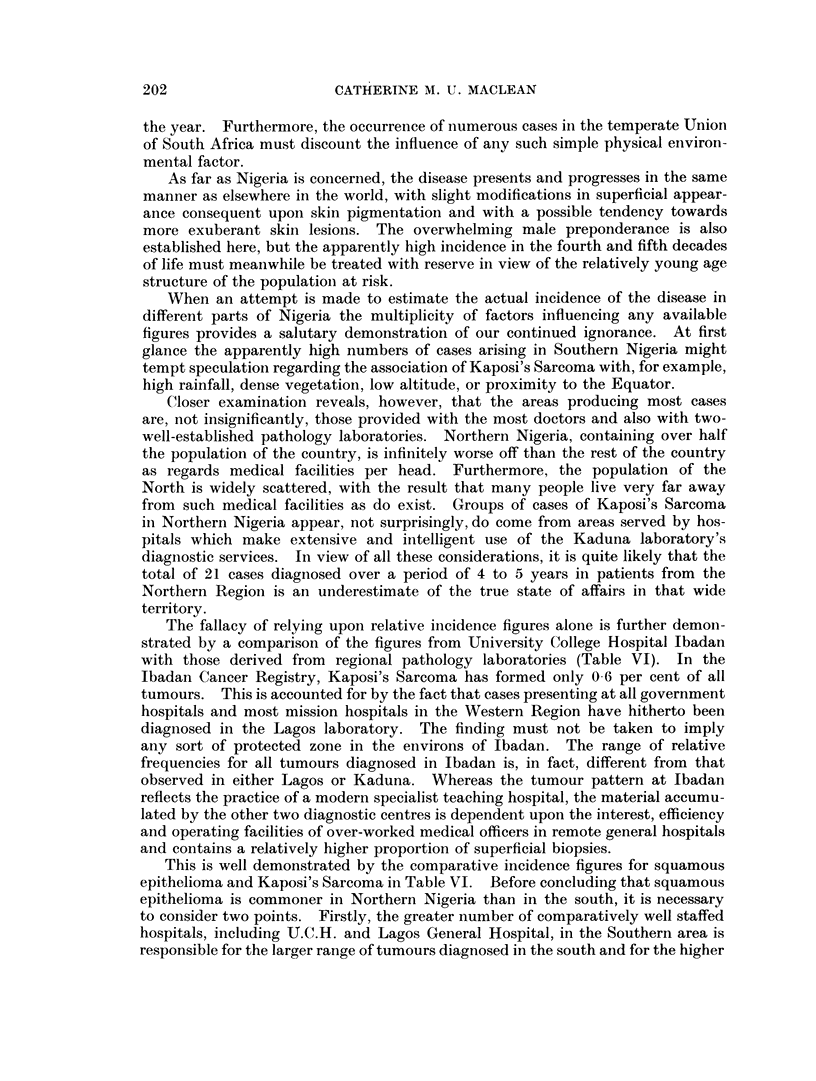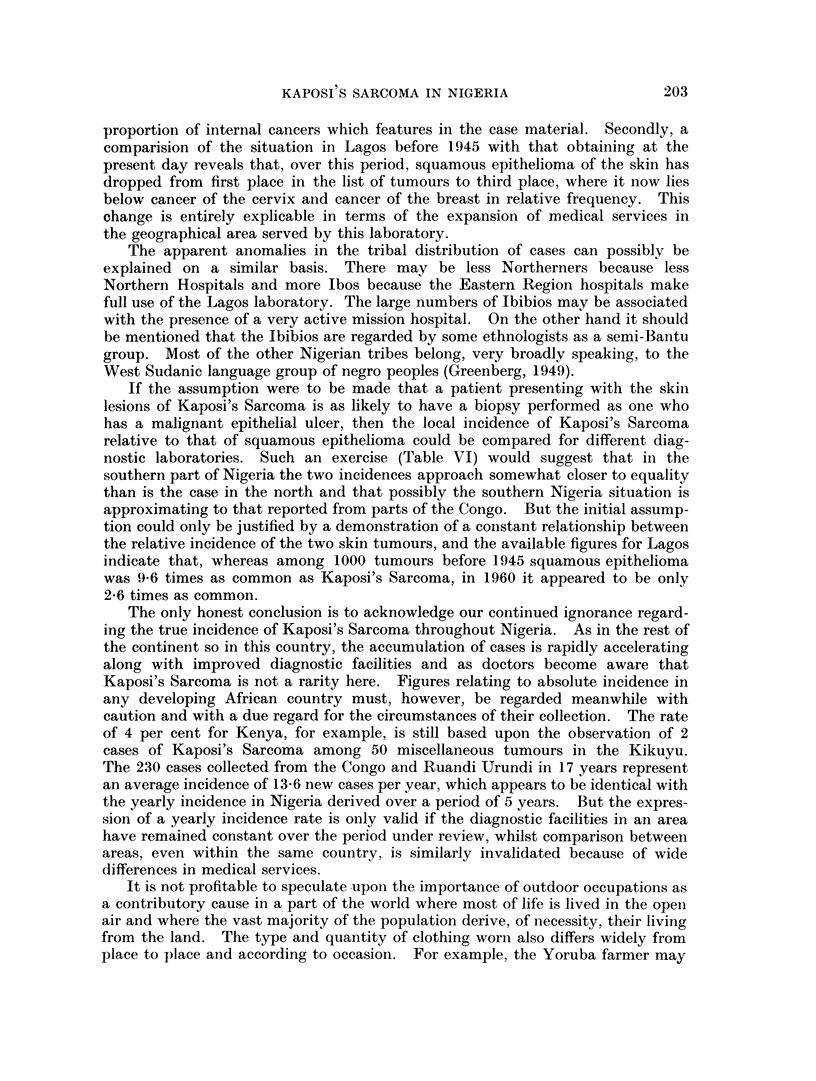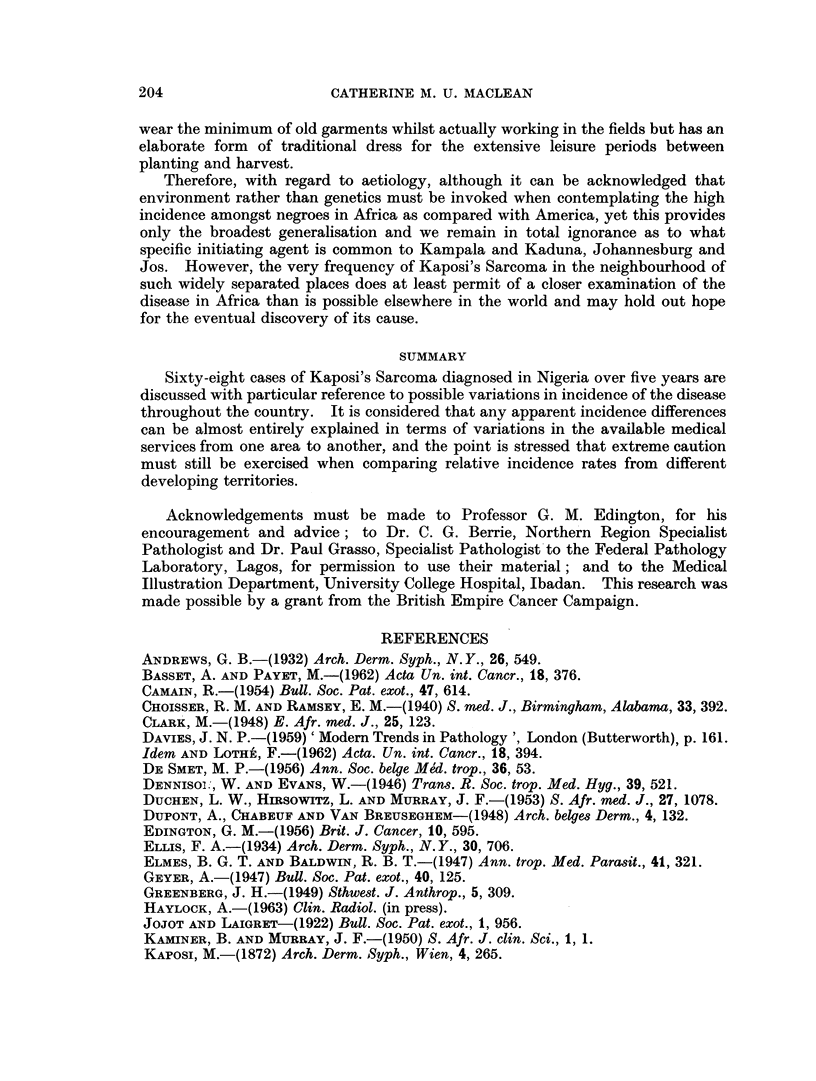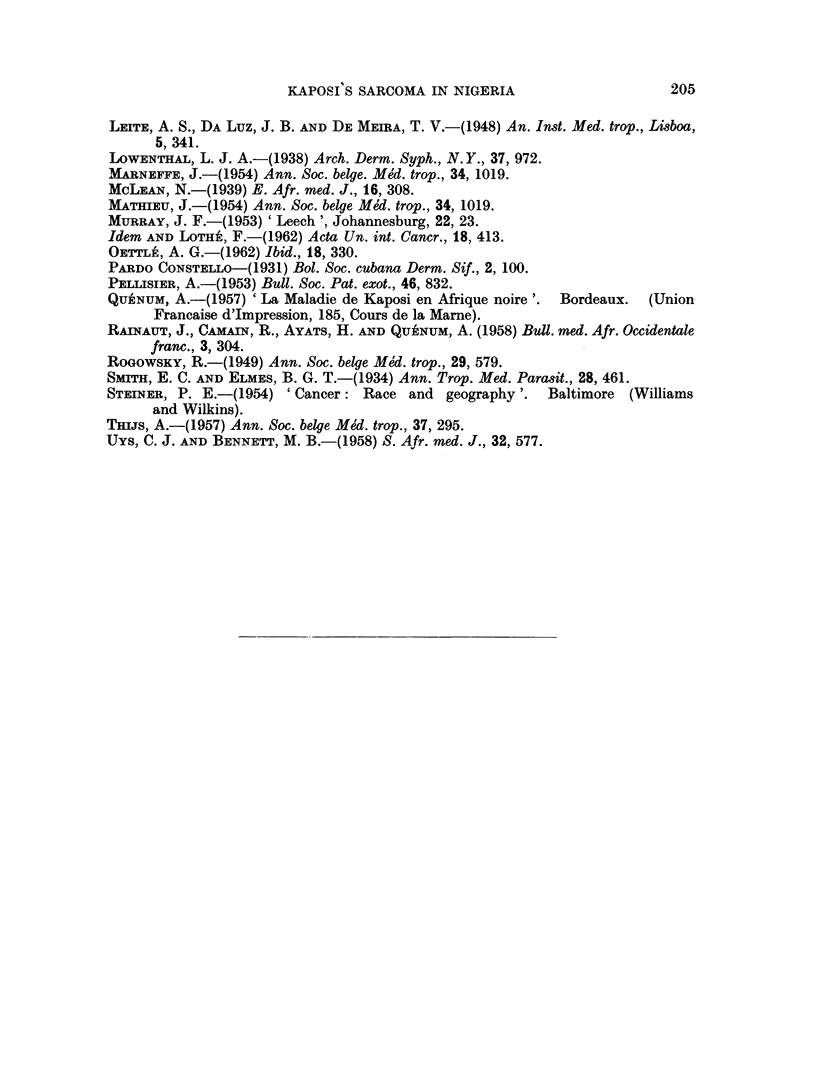# Kaposi's Sarcoma in Nigeria

**DOI:** 10.1038/bjc.1963.28

**Published:** 1963-06

**Authors:** Catherine M. U. Maclean


BRITISH JOURNAL OF CANCER

VOL. XVII JUNE, 1963 NO. 2

### KAPOSI'S SARCOMA IN NIGERIA

CATHERINE M. U. MACLEAN

*From the Ibadan Cancer Registry, University College Hospital, Ibadan, Nigeria*

Received March 12, 1963

THE extent of the literature which has appeared on the subject of Kaposi's Sarcoma during the ninety years since Kaposi first described, from Vienna in 1872, the condition which he called " idiopathic multiple pigment sarcoma ", has been indicative not only of the ambiguous histological status and dubious aetiology of this disease but also of its essential rarity. When, after 68 years, Choisser and Ramsey (1940) reviewed the European and American literature, they found reports of 600 cases. In reporting the first African case from the French Cameroons in 1922, Jojot and Laigret were impressed not so much by the discovery of this particular form of malignant disease as by the demonstration which the case afforded that cancer did occur at all in Africa, " It is a diagnosis ", they observed, " which, on the whole, one does not consider among the black inhabitants of this region ". The large numbers of African cases of Kaposi's Sarcoma which have been collected since the time this cautious statement was made are summarised in Table I.

Commenting upon the pattern of distribution of the disease which appeared to evolve from the African experience, Quénum (1957) suggested that the incidence of Kaposi's Sarcoma increased in proximity to the Equator. Davies (1959) went further when he remarked, " It is comparatively rare in the dry sandy areas but commoner in the moister tropical areas and also on the upland plateaux ". The search for an environmental influence was continued by Oettlé (1962) who statistically analysed different African reports in terms of the relative frequency of Kaposi's Sarcoma amongst tumours from various territories. He referred to the low frequency in Ghana, Rhodesia and French West Africa (below 1 per cent), compared successively with the Union of South Africa (0·8–2 per cent), Nigeria (2–3 per cent), Kenya and Uganda (4–5 per cent) and with the high frequency in parts of the Congo (4·3–10·4 per cent).

The present paper is concerned with the characteristics, incidence and geographic distribution throughout Nigeria of 68 cases of Kaposi's Sarcoma diagnosed in this country during the past five years.

*Collection of data*

The Ibadan Cancer Registry has, since early 1960, collected information relating to all cases of malignant disease diagnosed in this city. Two-thirds of the

TABLE I.—*Kaposi's Sarcoma in Africa* 1922–62*

| Author | Period | Place | Total cases reported |
|---|---|---|---|
| Jojot and Laigret (1922) | 1922 | Cameroon | 1 |
| Smith and Elmes (1934) | 1926–33 | Lagos, Nigeria | 10 |
| Lowenthal (1938) | 1938 | Uganda | 1 |
| Mclean (1939) | 1939 | Kenya | 2 |
| Dennison and Evans (1946) | 1946 | Nigeria | 1 |
| Elmes and Baldwin (1947) | 1934–45 | Lagos | 24 |
| Geyer (1947) | 1947 | Dakar | 2 |
| Clark (1948) | 1948 | Kenya | 2 |
| Dupont *et al.* (1948) | 1948 | Cameroon | 2 |
| Mathieu (1954) | 1948 | Belg. Congo | 1 |
| Edington (1956) | 1923–55 | Gold Coast | 10 |
| Rogowsky (1949) | 1949 | Belg. Congo | 1 |
| Leite *et al.* (1948) | 1948 | Angola | 1 |
| De Smet (1956) | 1945–55 | Belg. Congo | 12 |
| Camain (1954) | 1942–53 | Dakar | 7 (including two Europeans) |
| Thijs (1957) | 1939–55 | Belg. Congo and Ruanda Urundi | 230 |
| Pelliser (1953) | before 1953 | Brazzaville | 18 |
| Duchen *et al.* (1953) | 1953 | S. Africa | 1 |
| Marneffe (1954) | before 1954 | Urundi | 4 |
| Uys and Bennet (1958) | 1958 | Cape Town, S.A. | 1 (Coloured S.A.) |
| Rainaut *et al.* (1958) | 1958 | Dakar | 1 |
| Basset and Payet (1962) | 1959–61 | Dakar | 5 |
| Murray and Lothé (1962) | 1942–60<br>1934–61 | S. Africa<br>Uganda | 496 (483 Africans) |
| Maclean (this paper) | 1958–62 | Nigeria | 68 |

* References to individual cases and to some small groups of cases subsequently included in larger series from the same territory have been purposely omitted from this list, which is not intended to enumerate all the publications on Kaposi's Sarcoma in Africa.

actual patients come from beyond the city of Ibadan but the great majority (over 80 per cent) are Yorubas, the dominant tribe of the Western Region of Nigeria. On looking into the tribal distribution of eleven cases of Kaposi's Sarcoma which had been registered in the period 1960–1962, it was discovered that only three of these cases had been Yorubas. It seemed worth while extending the series to include cases diagnosed over a longer period and in other parts of Nigeria in order to consider whether there was any unusual distribution of the disease throughout the extent of this large territory.

### *Available sources of material*

Although patients with malignant disease may travel a considerable distance to Ibadan for treatment, the hospital is designed primarily as a Federal teaching institution and its laboratory facilities are not intended to be used as a diagnostic centre for this Region of Nigeria. Until recently, when a Specialist Pathologist was appointed to the Government General Hospital in Ibadan, doctors in the Western Region were obliged to send material to the Federal laboratory services in Lagos for specialist opinion on histology. Nevertheless, five or six mission hospitals in the Western Region have, by informal arrangement, become accustomed to sending histological material to the University College Hospital from time to time.

The Federal Pathology Laboratory in Lagos collects histological material from the Eastern Region, the Federal Territory of Lagos and, until about a year ago, from the Western Region also. The specimens are derived from both

Government and mission hospitals. By kind consent of the Federal Specialist Pathologist it was possible to examine the files for information and slides relating to cases of Kaposi's Sarcoma diagnosed during the four year period from 1958–1961 and to enumerate the total cases of malignant disease diagnosed in Lagos in one of those years.

The Northern Regional Pathology Laboratory has been sited in Kaduna, the administrative capital, since 1959. To this laboratory are sent histological specimens from all over Northern Nigeria. A pathologist has sometimes been stationed in Kano where he deals with material from the city hospital only. The Northern Region Specialist Pathologist kindly allowed access to files and histological material for the period 1959–62. An enumeration of total tumours for one of these years was also made.

There are 84 government general hospitals and 59 general missionary hospitals in Nigeria. Over half the government hospitals are in the charge of one doctor. The position of the mission hospitals is slightly better, only 40 per cent of them having to depend upon a single doctor. The hospitals also vary widely in respect of their bed numbers. Reply-paid questionnaires were sent to 77 mission and government general hospitals of over 90 beds throughout Nigeria (excluding those in regular contact with U.C.H.) enquiring after possible cases of Kaposi's Sarcoma diagnosed during the previous five years. Thirty-seven replies were received and by this means a few extra cases were discovered which had been diagnosed at other hospitals. From one hospital material had been sent to London for opinion. Careful cross checking was necessary to avoid duplication, as in the case of a man from the Niger Delta area, who was reported on the questionnaire from the parent hospital, from whom specimens were thrice sent to Lagos and who eventually turned up as an in-patient in U.C.H. in 1961.

Table II analyses the sources of the case material obtained in this extended search.

TABLE II.—*The Sources of* 68 *Diagnoses of Kaposi's Sarcoma*

| Place | Period | Number of cases |
|---|---|---|
| Ibadan | 1958–1962 | 17 |
| Lagos | 1958–1961 | 25 |
| Kaduna | 1959–1962 | 16 |
| Other hospitals (All Regions) | 1958–1962 | 10 |
| | Total | 68 |

*Clinical features*

Although all the cases had been confirmed by biopsy, the clinical information was deficient in certain respects and was only complete for the Ibadan cases.

In these latter the mode of presentation of the disease in adults did not differ from that reported from elsewhere in Africa, being characterized by vegetating, vascular nodules occurring predominantly on the extremities and frequently accompanied by oedema of the limb. Pain was minimal, although disability was progressive as the disease process extended. One case, however, appeared to have undergone considerable regression over a period of five years, without specific therapy ; the legs showed eventually a woody induration and some hard nodules about the ankle. Autopsy performed on a male aged 60 who died in U.C.H. demonstrated the only proved instance of visceral involvement in the series. There was widespread involvement of heart, trachea, paratracheal nodes and lesions in the lower lobe of one lung, as well as skin nodules on the legs. No case of childhood Kaposi's Sarcoma, featuring massive involvement of lymphatic nodes and with lesions on the eyelids or conjunctivae, as described from Uganda (Davies and Lothé, 1962), has been seen in Ibadan. The youngest case here, a boy of 14, presented with indurated plaques along the course of a saphenous vein in a swollen limb. Among the Kaduna records was one instance of a haemangio-endothelioma of the eyelid in a child aged 3 which might possibly have been Kaposi's Sarcoma. There were, however, no supplementary clinical details nor had any lymph node been biopsied.

Haylock (1963) has described the radiographic findings, including angiography, in a group of Ibadan cases and has referred to the results of intra-arterial mustine therapy.

TABLE III.—*The Tribal Origin of Kaposi's Sarcoma Cases Compared With the Tribal Composition of the Nigerian Population*

| Tribe | Number of K.S. cases | Percentage of K.S. cases | Percentage of total population |
|---|---|---|---|
| Ibo | 20 | 29·4 | 17·4 |
| Main Northern Tribes | 12 | 17·7 | 34·8 |
| Yoruba | 11 | 16·2 | 16·2 |
| Ibibio | 6 | 8·8 | 2·5 |
| Edo | 5 | 7·3 | 1·6 |
| Unspecified Tribes | 14 | 20·6 | 26·5 |
| Non Nigerians | 0 | 0 | 0·1 |

Over the whole Nigerian series the proportion of males was 89 per cent (60 cases). Three of the 11 Yoruba cases were females and the only case from the Southern Cameroons was a woman.

The age of the patients, where this was known, is given in Table IV. The remainder were simply cited as " adult ".

TABLE IV.—*The Ages of* 45 *Cases of Kaposi's Sarcoma*

| Age group | 0–14 | 15–19 | 20–24 | 25–29 | 30–34 | 35–39 | 40–44 | 45–49 | 50–54 | 55–59 | 60–64 |
|---|---|---|---|---|---|---|---|---|---|---|---|
| Number of cases | 3 | 2 | 3 | 5 | 3 | 6 | 9 | 5 | 4 | 3 | 2 |

In 64 instances the site from which the histological specimen was taken was specified. Although this information is not sufficient to assess what may have been the total clinical extent of the disease in each case, it is possible to derive some impression of the relative frequency of involvement of different parts of the body. The data is summarised in Table V.

TABLE V.—*The Site of Lesion in* 64 *Cases of Kaposi's Sarcoma*

| Site | Number of times affected |
|---|---|
| Lower limbs, one or both | 47 |
| Upper limbs, one or both | 12 |
| Head and neck | 6 |
| Trunk | 5 |
| Penis and scrotum | 5 |
| Lymph nodes | 2 |
| Viscera | 1 |

*Histology*

The lesions took the form of proliferating bundles and whorls of pale spindle cells, not markedly malignant in appearance, interspersed with narrow blood-filled spaces. The thin-walled vessels were of varying capacity and the proportion of vascular to spindle-celled elements varied from case to case.

In some instances the dense nuclei and mitotic figures of widely ramifying spindle cells gave an impression of very active growth, haemorrhage was present and an admixture of inflammatory cells. In other case lacunae-like blood spaces lay in a tissue composed of much looser strands of spindle cells. The appearance of a fibrous capsule and thrombosis in the blood vessels seems to have been associ-

FIG. 1.—To illustrate the distribution of Kaposi's Sarcoma throughout Nigeria (1958–62).

ated with clinical regregression of the lesions. Successive biopsies from the same patient varied as much as biopsies from different subjects, but the main features of the disease were still clearly recognisable.

*Occupation*

The majority of the Ibadan cases were peasant farmers and artisans, but one was a Civil Servant, one an electrical generator operator and the youngest was a school boy. The case already mentioned whose lesions were regressing was a man of Southern Nigerian origin who had spent 20 years touring Northern Nigeria on geological surveys.

*Geographical distribution of cases*

In 63 out of these 68 cases the geographical location of the patients was known and it was consequently possible to plot their origin on the map in Fig. 1. From the Kaduna series there were in addition one Hausa (Northerner), whose residence was not precisely specified, and two Ibos. The Ibos, from over-crowded Eastern Nigeria, frequently find temporary employment in the North. It would be safe to assume that the Eastern Region was the home of one further Ibo male in the Lagos series whose origin was not given. One case came from the Southern Cameroons, which is no longer part of Nigeria.

*Tribal distribution of cases*

The tribal origins of the Nigerian cases of Kaposi's Sarcoma have been compared in Table III, with the proportions of different tribes in the population as a whole (1952–53 Nigerian Census). It will be noted that Northern tribes appear to be under represented whilst there are, on the other hand, proportionately more Ibos, Ibibios and Edos than in the general population. The figures for Yorubas in both groups coincide. The possible significance of these findings will be discussed later.

*Incidence throughout Nigeria*

Elmes and Baldwin, in 1947, found that Kaposi's Sarcoma had accounted for 2·4 per cent of 1000 tumours diagnosed in Lagos over the previous 10 years. During 3 years in Ibadan the relative frequency of Kaposi's Sarcoma among 1934 tumours was 0·6 per cent ; in Lagos in 1960 the frequency was 3·6 per cent, whilst in Kaduna in 1962 it was 2·6 per cent. These figures are summarised in Table VI, which also indicates the corresponding incidence of squamous epithelioma

TABLE VI.—*The Relative Incidence of Kaposi's Sarcoma and Squamous Epithelioma of the Skin throughout Nigeria*

| | | | Kaposi's sarcoma | | Squamous epithelioma skin | |
|---|---|---|---|---|---|---|
| Place | Period | Total tumours | No. of cases | Total tumours per cent | No. of cases | Total tumours per cent |
| Lagos | 1935–44 | 1000 | 24 | 2·4 | 231 | 23·1 |
| Lagos | 1960 | 358 | 13 | 3·6 | 33 | 9·2 |
| Kaduna | 1962 | 395 | 10 | 2·6 | 55 | 13·9 |
| Ibadan | 1960–62 | 1934 | 11 | 0·6 | 45 | 2·3 |

at each centre. The reason for making such a comparison is that, from part of the Congo, Thijs (1957) observed that Kaposi's Sarcoma was as common as squamous epithelioma, whilst it is the commonest skin tumour among Bantu in the Transvaal (Oettlé, 1962).

It is important to take cognisance of the proportion of total tumours which tumours of the skin represent in different centres. For example, in Lagos up to 1945, skin tumours, excluding malignant melanoma, were the commonest form of malignant disease recorded. At the present day, however, they feature as rather less common (9·5 per cent) than carcinoma of the cervix and breast (each 10 per cent). The three commonest forms of malignant disease in the Ibadan Cancer Registry at present are carcinoma of the cervix, childhood lymphoma and carcinoma of the breast.

*The medical services*

The number of doctors relative to the population throughout Nigeria has a bearing on the present study. For example, although over 20 million people, at least 60 per cent of the population of Nigeria, live in the Northern Region yet they are at present served by only 200 doctors. Table VI compares the different

TABLE VII.—*The Doctor : Patient Ratio in Different Parts of Nigeria*

| Region | Doctor patient ratio |
|---|---|
| Northern Region . | 1 per 100,000 |
| Western Region . | 1 per 25,000 |
| Eastern Region . | 1 per 46,000 |
| Federal Territory of Lagos . | 1 per 2,000 |

regions in respect of doctor patient ratios. Ibadan, being the site of U.C.H. has, like Lagos, a high doctor patient ratio, which has the effect of raising the figure for the Western Region as a whole.

*Climate*

Northern Nigeria benefits for part of the year from the " Harmattan ", a dry wind which blows off the Sahara. Although day temperatures are on the average higher than in the south, these are relieved by cold at night throughout most of the year and in the extreme north-east the temperature sometimes drops below freezing point. By contrast, Southern Nigeria, particularly the forested zone, is characterised by very high humidity associated with incessant heat. The combination of heat and humidity is most marked along the coastal belt of swamps which extends both east and west from the Niger Delta area.

### DISCUSSION

Only a few individual case reports of Kaposi's Sarcoma amongst negroes in the new world have been published. Pardo Costello (1931) reported a case in a Cuban negro, Andrews (1932) described the disease in a mulatto boy from Chicago and Ellis (1934) reported a further case in an American negro. Later American figures were reviewed by Steiner (1954), who was confident that the disease was much less common amongst negroes than amongst whites in the United States. In the parent continent, on the other hand, the position is completely reversed. It has been demonstrated in South Africa that the disease is twenty times as common in the Bantu as in the white inhabitants of the Union (Murray, 1953 ; Murray and Lothé, 1962). A particularly high incidence has been observed in the Congo. Experience in Nigeria now shows that it is no rarity in this portion of the old Slave Coast. It is clear, therefore, that those territories from which the ancestors of today's American negroes were derived contain some factor whch provokes or encourages the development of this mysterious condition.

It is impossible to implicate climatic conditions alone. The disease in Africa occurs throughout a wide range of territories which vary enormously in both temperature and humidity. Countries like Uganda and the Congo both span the Equator, yet the former enjoys a relatively temperate climate, whereas the latter is uniformly hot and, in the Equatorial region, rain falls almost daily throughout the year. Furthermore, the occurrence of numerous cases in the temperate Union of South Africa must discount the influence of any such simple physical environmental factor.

As far as Nigeria is concerned, the disease presents and progresses in the same manner as elsewhere in the world, with slight modifications in superficial appearance consequent upon skin pigmentation and with a possible tendency towards more exuberant skin lesions. The overwhelming male preponderance is also established here, but the apparently high incidence in the fourth and fifth decades of life must meanwhile be treated with reserve in view of the relatively young age structure of the population at risk.

When an attempt is made to estimate the actual incidence of the disease in different parts of Nigeria the multiplicity of factors influencing any available figures provides a salutary demonstration of our continued ignorance. At first glance the apparently high numbers of cases arising in Southern Nigeria might tempt speculation regarding the association of Kaposi's Sarcoma with, for example, high rainfall, dense vegetation, low altitude, or proximity to the Equator.

Closer examination reveals, however, that the areas producing most cases are, not insignificantly, those provided with the most doctors and also with two-well-established pathology laboratories. Northern Nigeria, containing over half the population of the country, is infinitely worse off than the rest of the country as regards medical facilities per head. Furthermore, the population of the North is widely scattered, with the result that many people live very far away from such medical facilities as do exist. Groups of cases of Kaposi's Sarcoma in Northern Nigeria appear, not surprisingly, do come from areas served by hospitals which make extensive and intelligent use of the Kaduna laboratory's diagnostic services. In view of all these considerations, it is quite likely that the total of 21 cases diagnosed over a period of 4 to 5 years in patients from the Northern Region is an underestimate of the true state of affairs in that wide territory.

The fallacy of relying upon relative incidence figures alone is further demonstrated by a comparison of the figures from University College Hospital Ibadan with those derived from regional pathology laboratories (Table VI). In the Ibadan Cancer Registry, Kaposi's Sarcoma has formed only 0·6 per cent of all tumours. This is accounted for by the fact that cases presenting at all government hospitals and most mission hospitals in the Western Region have hitherto been diagnosed in the Lagos laboratory. The finding must not be taken to imply any sort of protected zone in the environs of Ibadan. The range of relative frequencies for all tumours diagnosed in Ibadan is, in fact, different from that observed in either Lagos or Kaduna. Whereas the tumour pattern at Ibadan reflects the practice of a modern specialist teaching hospital, the material accumulated by the other two diagnostic centres is dependent upon the interest, efficiency and operating facilities of over-worked medical officers in remote general hospitals and contains a relatively higher proportion of superficial biopsies.

This is well demonstrated by the comparative incidence figures for squamous epithelioma and Kaposi's Sarcoma in Table VI. Before concluding that squamous epithelioma is commoner in Northern Nigeria than in the south, it is necessary to consider two points. Firstly, the greater number of comparatively well staffed hospitals, including U.C.H. and Lagos General Hospital, in the Southern area is responsible for the larger range of tumours diagnosed in the south and for the higher proportion of internal cancers which features in the case material. Secondly, a comparision of the situation in Lagos before 1945 with that obtaining at the present day reveals that, over this period, squamous epithelioma of the skin has dropped from first place in the list of tumours to third place, where it now lies below cancer of the cervix and cancer of the breast in relative frequency. This change is entirely explicable in terms of the expansion of medical services in the geographical area served by this laboratory.

The apparent anomalies in the tribal distribution of cases can possibly be explained on a similar basis. There may be less Northerners because less Northern Hospitals and more Ibos because the Eastern Region hospitals make full use of the Lagos laboratory. The large numbers of Ibibios may be associated with the presence of a very active mission hospital. On the other hand it should be mentioned that the Ibibios are regarded by some ethnologists as a semi-Bantu group. Most of the other Nigerian tribes belong, very broadly speaking, to the West Sudanic language group of negro peoples (Greenberg, 1949).

If the assumption were to be made that a patient presenting with the skin lesions of Kaposi's Sarcoma is as likely to have a biopsy performed as one who has a malignant epithelial ulcer, then the local incidence of Kaposi's Sarcoma relative to that of squamous epithelioma could be compared for different diagnostic laboratories. Such an exercise (Table VI) would suggest that in the southern part of Nigeria the two incidences approach somewhat closer to equality than is the case in the north and that possibly the southern Nigeria situation is approximating to that reported from parts of the Congo. But the initial assumption could only be justified by a demonstration of a constant relationship between the relative incidence of the two skin tumours, and the available figures for Lagos indicate that, whereas among 1000 tumours before 1945 squamous epithelioma was 9·6 times as common as Kaposi's Sarcoma, in 1960 it appeared to be only 2·6 times as common.

The only honest conclusion is to acknowledge our continued ignorance regarding the true incidence of Kaposi's Sarcoma throughout Nigeria. As in the rest of the continent so in this country, the accumulation of cases is rapidly accelerating along with improved diagnostic facilities and as doctors become aware that Kaposi's Sarcoma is not a rarity here. Figures relating to absolute incidence in any developing African country must, however, be regarded meanwhile with caution and with a due regard for the circumstances of their collection. The rate of 4 per cent for Kenya, for example, is still based upon the observation of 2 cases of Kaposi's Sarcoma among 50 miscellaneous tumours in the Kikuyu. The 230 cases collected from the Congo and Ruandi Urundi in 17 years represent an average incidence of 13·6 new cases per year, which appears to be identical with the yearly incidence in Nigeria derived over a period of 5 years. But the expression of a yearly incidence rate is only valid if the diagnostic facilities in an area have remained constant over the period under review, whilst comparison between areas, even within the same country, is similarly invalidated because of wide differences in medical services.

It is not profitable to speculate upon the importance of outdoor occupations as a contributory cause in a part of the world where most of life is lived in the open air and where the vast majority of the population derive, of necessity, their living from the land. The type and quantity of clothing worn also differs widely from place to place and according to occasion. For example, the Yoruba farmer may wear the minimum of old garments whilst actually working in the fields but has an elaborate form of traditional dress for the extensive leisure periods between planting and harvest.

Therefore, with regard to aetiology, although it can be acknowledged that environment rather than genetics must be invoked when contemplating the high incidence amongst negroes in Africa as compared with America, yet this provides only the broadest generalisation and we remain in total ignorance as to what specific initiating agent is common to Kampala and Kaduna, Johannesburg and Jos. However, the very frequency of Kaposi's Sarcoma in the neighbourhood of such widely separated places does at least permit of a closer examination of the disease in Africa than is possible elsewhere in the world and may hold out hope for the eventual discovery of its cause.

### SUMMARY

Sixty-eight cases of Kaposi's Sarcoma diagnosed in Nigeria over five years are discussed with particular reference to possible variations in incidence of the disease throughout the country. It is considered that any apparent incidence differences can be almost entirely explained in terms of variations in the available medical services from one area to another, and the point is stressed that extreme caution must still be exercised when comparing relative incidence rates from different developing territories.

Acknowledgements must be made to Professor G. M. Edington, for his encouragement and advice; to Dr. C. G. Berrie, Northern Region Specialist Pathologist and Dr. Paul Grasso, Specialist Pathologist to the Federal Pathology Laboratory, Lagos, for permission to use their material; and to the Medical Illustration Department, University College Hospital, Ibadan. This research was made possible by a grant from the British Empire Cancer Campaign.

### REFERENCES

Andrews, G. B.—(1932) *Arch. Derm. Syph., N.Y.*, **26**, 549.
Basset, A. and Payet, M.—(1962) *Acta Un. int. Cancr.*, **18**, 376.
Camain, R.—(1954) *Bull. Soc. Pat. exot.*, **47**, 614.
Choisser, R. M. and Ramsey, E. M.—(1940) *S. med. J., Birmingham, Alabama*, **33**, 392.
Clark, M.—(1948) *E. Afr. med. J.*, **25**, 123.
Davies, J. N. P.—(1959) 'Modern Trends in Pathology', London (Butterworth), p. 161.
*Idem* and Lothé, F.—(1962) *Acta. Un. int. Cancr.*, **18**, 394.
De Smet, M. P.—(1956) *Ann. Soc. belge Méd. trop.*, **36**, 53.
Dennison, W. and Evans, W.—(1946) *Trans. R. Soc. trop. Med. Hyg.*, **39**, 521.
Duchen, L. W., Hirsowitz, L. and Murray, J. F.—(1953) *S. Afr. med. J.*, **27**, 1078.
Dupont, A., Chabeuf and Van Breuseghem—(1948) *Arch. belges Derm.*, **4**, 132.
Edington, G. M.—(1956) *Brit. J. Cancer*, **10**, 595.
Ellis, F. A.—(1934) *Arch. Derm. Syph., N.Y.*, **30**, 706.
Elmes, B. G. T. and Baldwin, R. B. T.—(1947) *Ann. trop. Med. Parasit.*, **41**, 321.
Geyer, A.—(1947) *Bull. Soc. Pat. exot.*, **40**, 125.
Greenberg, J. H.—(1949) *Sthwest. J. Anthrop.*, **5**, 309.
Haylock, A.—(1963) *Clin. Radiol.* (in press).
Jojot and Laigret—(1922) *Bull. Soc. Pat. exot.*, **1**, 956.
Kaminer, B. and Murray, J. F.—(1950) *S. Afr. J. clin. Sci.*, **1**, 1.
Kaposi, M.—(1872) *Arch. Derm. Syph., Wien*, **4**, 265.

LEITE, A. S., DA LUZ, J. B. AND DE MEIRA, T. V.—(1948) *An. Inst. Med. trop., Lisboa*, **5**, 341.
LOWENTHAL, L. J. A.—(1938) *Arch. Derm. Syph., N.Y.*, **37**, 972.
MARNEFFE, J.—(1954) *Ann. Soc. belge. Méd. trop.*, **34**, 1019.
McLEAN, N.—(1939) *E. Afr. med. J.*, **16**, 308.
MATHIEU, J.—(1954) *Ann. Soc. belge Méd. trop.*, **34**, 1019.
MURRAY, J. F.—(1953) ' Leech ', Johannesburg, **22**, 23.
*Idem* AND LOTHÉ, F.—(1962) *Acta Un. int. Cancr.*, **18**, 413.
OETTLÉ, A. G.—(1962) *Ibid.*, **18**, 330.
PARDO CONSTELLO—(1931) *Bol. Soc. cubana Derm. Sif.*, **2**, 100.
PELLISIER, A.—(1953) *Bull. Soc. Pat. exot.*, **46**, 832.
QUÉNUM, A.—(1957) ' La Maladie de Kaposi en Afrique noire '. Bordeaux. (Union Francaise d'Impression, 185, Cours de la Marne).
RAINAUT, J., CAMAIN, R., AYATS, H. AND QUÉNUM, A. (1958) *Bull. med. Afr. Occidentale franc.*, **3**, 304.
ROGOWSKY, R.—(1949) *Ann. Soc. belge Méd. trop.*, **29**, 579.
SMITH, E. C. AND ELMES, B. G. T.—(1934) *Ann. Trop. Med. Parasit.*, **28**, 461.
STEINER, P. E.—(1954) ' Cancer : Race and geography '. Baltimore (Williams and Wilkins).
THIJS, A.—(1957) *Ann. Soc. belge Méd. trop.*, **37**, 295.
UYS, C. J. AND BENNETT, M. B.—(1958) *S. Afr. med. J.*, **32**, 577.